# Pattern of Ambulatory Care Visits to Obstetrician-Gynecologists in Taiwan: A Nationwide Analysis

**DOI:** 10.3390/ijerph120606832

**Published:** 2015-06-16

**Authors:** An-Min Lynn, Li-Jung Lai, Ming-Hwai Lin, Tzeng-Ji Chen, Shinn-Jang Hwang, Peng-Hui Wang

**Affiliations:** 1 Department of Family Medicine, Taipei Veterans General Hospital, No. 201, Sec. 2, Shi-Pai Road, Taipei 112, Taiwan; E-Mails: nashsaka@hotmail.com (A.-M.L.); larena_lai@yahoo.com (L.-J.L.); tjchen@vghtpe.gov.tw (T.-J.C.); sjhwang@vghtpe.gov.tw (S.-J.H.); 2 School of Medicine, National Yang-Ming University, No.155, Sec. 2, Linong Street, Taipei 112, Taiwan; E-Mail: phwang@vghtpe.gov.tw; 3 Department of Obstetrics and Gynecology, Taipei Veterans General Hospital, No. 201, Sec. 2, Shi-Pai Road, Taipei 112, Taiwan

**Keywords:** obstetrician-gynecologists, national health insurance, ambulatory visits

## Abstract

Although obstetrician-gynecologists (OB-GYNs) are the main actors in the provision of health care to women, their practice patterns have rarely been analyzed. The current study investigated the nationwide ambulatory visits to OB-GYNs in Taiwan using the National Health Insurance Research Database. From the 1/500 sampling datasets indicating 619,760 ambulatory visits in 2012, it was found that 5.8% (n = 35,697) of the visits were made to OB-GYNs. Two-fifths of the services provided were performed by male OB-GYNs aged 50–59 years. Women of childbearing age accounted for more than half of the visits to OB-GYNs (57.2%), and elderly patients above 60 years accounted for only 7.7%. The most frequent diagnoses were menstrual disorders and other forms of abnormal bleeding from the female genital tract (13.1%). Anti-infective agents were prescribed in 15.1% of the visits to OB-GYNs. The study revealed the proportion of aging practicing OB-GYNs, and our detailed results could contribute to evidence-based discussions on health policymaking.

## 1. Introduction

Obstetrician-gynecologists (OB-GYNs) play a fundamental role in women’s health, from delivering children and providing preventive health care services to treating gynecologic diseases. The progress of this specialty over the past few decades has contributed to the improvement of public health around the world. Although obstetrics and gynecology have been well established as major health care disciplines, the shortage and aging of practicing OB-GYNs have been noted globally [1,2,3]. The decreasing birth rate, insufficient reimbursement, and malpractice risk may be some of the factors exacerbating the difficulty in attracting new recruits [4]. Comprehensive information about obstetric and gynecologic care is needed to facilitate the analysis of existing problems. In the United States, the findings of the National Ambulatory Medical Care Survey (NAMCS) and the National Hospital Ambulatory Medical Care Survey (NHAMCS) conducted by the Centers for Disease Control and Prevention were published in a series of reports indicating the number of ambulatory visits to OB-GYNs and depicting the national profile of women’s health care [5,6,7]. The Collaborative Ambulatory Research Network of the American Congress of Obstetricians and Gynecologists also provided substantial information and influential guidance for policymaking [8,9,10]. However, in most countries, including Taiwan, relevant literature is scarce.

The aim of the current study was to investigate the nationwide pattern of ambulatory visits to OB-GYNs as recorded by Taiwan’s National Health Insurance (NHI) system in 2012. Besides the ages of patients and physicians, the analyses also included the diagnoses, procedures, and medications prescribed during these visits. The findings may provide evidence useful in discussions on OB-GYNs’ residency training and health policymaking, and may provide a basis for drawing international comparisons.

## 2. Methods

### 2.1. Database 

In Taiwan, the NHI program, which started in 1995, provides comprehensive health care coverage to more than 99% of the country's residents. The National Health Insurance Administration of the Ministry of Health and Welfare has released all de-identified claims data dating back to 1999 for academic research in the form of the National Health Insurance Research Database (NHIRD; http://w3.nhri.org.tw/nhird/). 

### 2.2. Study Population

We conducted this descriptive and cross-sectional study by accessing the sampling files of the year 2012 (S_CD20120.DAT and S_OO20120.DAT of NHIRD). The dataset “CD” is a collection of all outpatient visit files, and the dataset “OO” contains the outpatient order files. These two sampling files, comprising a total of 619,760 medical records, were derived by a 0.2% sampling ratio from the CD and OO datasets for 2012, excluding dentistry and traditional Chinese medicine. Each record contained the patient’s identification number, sex, birth date, date of consultation, medical facility, the specialty of the consulting physician, and up to three diagnosis codes as defined by the International Classification of Diseases, Ninth Revision, Clinical Modification (ICD-9-CM).

From the sampling medical records, the details of 35,697 ambulatory visits to OB-GYNs were extracted and analyzed. The National Health Insurance Administration also offered a list of reimbursable drugs with additional coding in the Anatomical Therapeutic Chemical (ATC) classification system (http://www.whocc.no/atc_ddd_index/). The basic data of the contracted medical care institutions provided the status of accreditation: academic medical center, metropolitan hospital, local community hospital, or physician clinic. The diagnoses, procedures, and medications prescribed during the visits to facilities of various levels were analyzed.

### 2.3. Statistical Analysis

The programming software Perl version 5.20.2 was used for data processing, and the statistical software SPSS version 22.0 (IBM) was used for statistical analysis. Pearson’s χ^2^ test was used for group comparisons. A *p*-value < 0.05 (two-tailed) was considered statistically significant.

## 3. Results

Based on the sampling data, of the 619,760 ambulatory visits made in 2012, 5.8% (n = 35,697) were made to OB-GYNs—a discipline that ranked sixth among all physician specialties (Table 1). OB-GYNs also accounted for 3.2% of insurance claims, amounting to an estimated NT$309 billion in 2012.

**Table 1:** Number and percentage of ambulatory visits and claims, by specialty, in Taiwan, based on the 1/500 sampling datasets comprising 619,760 ambulatory visits in 2012.

Specialty	Number of visits (%)	Claims (%)
**Family medicine**	116,551 (18.8)	51,157,561 (8.3)
**Internal medicine**	70,615 (11.4)	42,542,879 (6.9)
**Otorhinolaryngology**	67,881 (11.0)	29,718,395 (4.8)
**Pediatrics**	60,717 (9.8)	31,574,986 (5.1)
**Ophthalmology**	37,692 (6.1)	25,422,291 (4.1)
**Obstetrics & Gynecology**	35,697 (5.8)	19,674,700 (3.2)
**All others**		
**Total**	619,760 (100)	618,119,592 (100)

**Figure 1:**
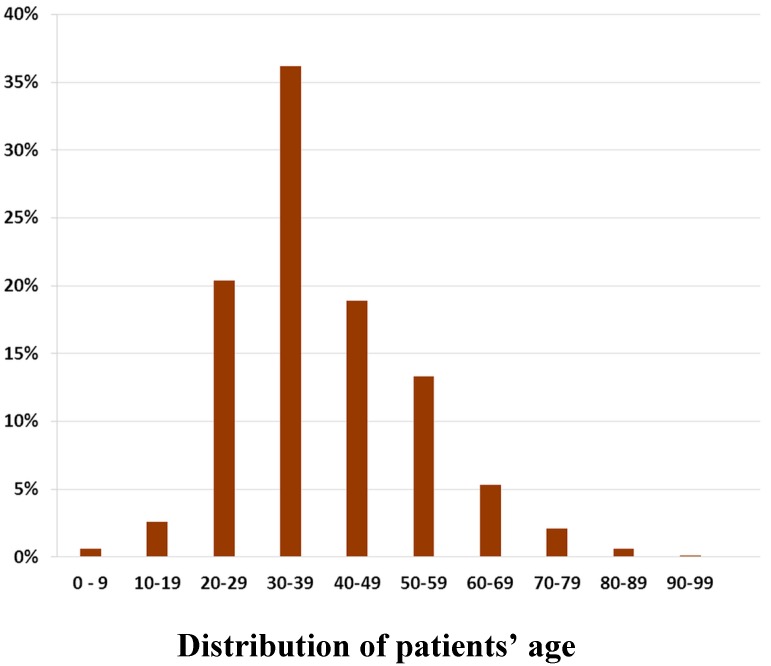
Number of ambulatory visits to OB-GYNs (n = 35,697) by patients’ age from the 1/500 sampling datasets comprising 619,760 ambulatory visits in 2012.

Among the ambulatory visits to OB-GYNs, 98.1% involved female patients, and only 1.9% were male patients. Stratifying the data by age group revealed that patients aged 30–39 years had the highest proportion (36.2%, n = 12,810) of ambulatory visits to OB-GYNs, followed by patients aged 20–29 years (20.4%, n = 7234). Female patients of around childbearing age accounted for more than half the total number of ambulatory visits to OB-GYNs (Figure 1).

**Figure 2:**
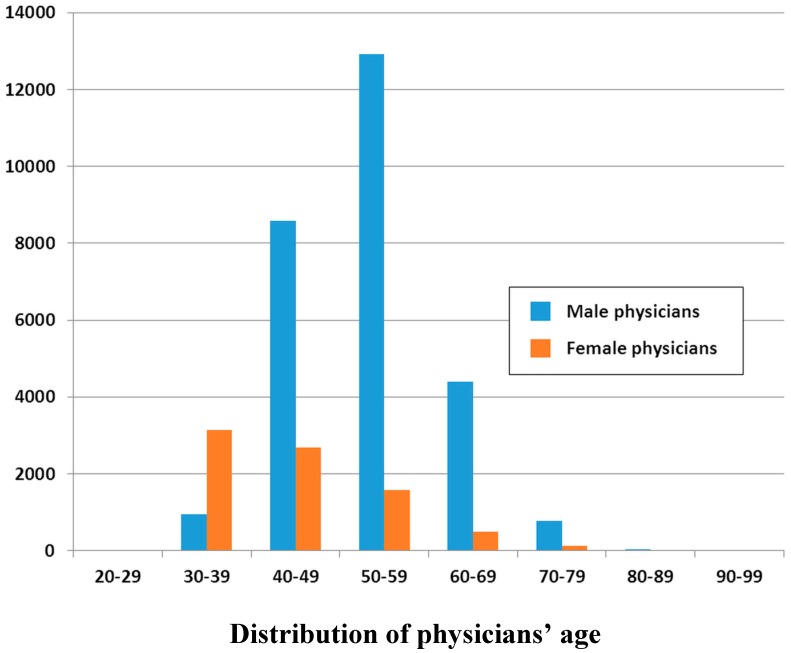
Number of ambulatory visits to OB-GYNs (n = 35,697) by physicians’ gender and age, from the 1/500 sampling datasets comprising 619,760 ambulatory visits in 2012.

The number of ambulatory visits to OB-GYNs is presented in Figure 2. It reveals that there are far fewer male physicians in the 30–39 years (n = 958) age range than in other working-age ranges, that is, 40–49 (n = 8577), 50–59 (n = 12,913), and 60–69 (n = 4405). The number of female physicians was the highest in the 30–39 years (n = 3,133) range compared with the other working-age ranges, even exceeding the number of male physicians in that age range. The ratio of female to male physicians was more than triple in the 30–39 age range (3133/958). 

In the current study, physician clinics remained the major ambulatory care providers and contributed to 61.6% (n = 21,974) of the ambulatory visits to OB-GYNs, followed by local community hospitals (15.0%), metropolitan hospitals (13.9%), and academic medical centers (9.5%). Among the ambulatory visits to OB-GYNs, 57.1% (n = 20,371) produced only one diagnosis. Analyzing the first diagnosis code in every medical record, we assembled a list of the top 10 most common diagnosis groups (Table 2). The top diagnosis was menstrual disorders and other abnormal bleeding from the female genital tract (13.1%), followed by normal pregnancy (12.4%), inflammatory disease of the cervix, vagina, and vulva (12.4%), special screening for malignant neoplasms (6.7%), and special investigations and examinations (4.7%). The ranking of the diagnosis groups varied according to hospital level. The “unknown” entry seemed to be the result of incorrect ICD-9 input or instances where claim actions were not successfully completed. Most of the records for physician clinics and the academic medical center did not have such mistakes.

**Table 2:** Number and percentage of ambulatory visits to OB-GYNs by disease group and hospital level in Taiwan, from the 1/500 sampling datasets comprising 619,760 ambulatory visits in 2012, top 10.

ICD-9CM *	Diagnosis group	Total N = 35,697 **	Academic medical center N = 3402	Metropolitan hospital N = 4945	Local community hospital N = 5365	Physician clinics N = 21,974
626	Disorders of menstruation	4667 (13.1)	129 (3.8)	340 (6.9)	617 (11.5)	3581 (16.3)
V22	Normal pregnancy	4443 (12.4)	617 (18.1)	1134 (22.9)	1065 (19.9)	1627 (7.4)
616	Inflammatory disease of the cervix, vagina, and vulva	4431 (12.4)	127 (3.7)	313 (6.3)	572 (10.7)	3419 (15.6)
V76	Special screening for malignant neoplasms	2374 (6.7)	312 (9.2)	744 (15.04)	485 (9.0)	823 (3.74)
V72	Special investigations & examinations	1677 (4.7)	179 (5.3)	176 (3.6)	207 (3.9)	1115 (5.1)
	Unknown	1559 (4.4)	0 (0.0)	5 (0.1)	1 (0.01)	1552 (7.1)
789	Other symptoms involving abdomen	1512 (4.2)	56 (1.6)	137 (2.8)	253 (4.7)	1066 (4.9)
627	Menopausal disorders	1293 (3.6)	180 (5.3)	326 (6.6)	242 (4.5)	545 (2.5)
614	Inflammatory disease of ovary, pelvic tissue	900 (2.5)	30 (0.88)	51 (1.0)	83 (1.5)	736 (3.3)
112	Candidiasis	676 (1.9)	11 (0.3)	27 (0.5)	82 (1.5)	556 (2.5)

* The International Classification of Diseases, 9th Revision, Clinical Modification. ** Eleven other ambulatory visits were served by midwife clinics.

Overall, the most common procedures performed during ambulatory visits to OB-GYNs were pelvic examination (19%, *n* = 6774), cervical pathology (12.7%, *n* = 4536), vaginal irrigation (11.4%, *n* = 4058), Pap smear sampling/pelvic examination (10.2%, *n* = 3653), gynecologic ultrasound (7.8%, *n* = 2802), and pregnancy test – enzyme immunoassays (6.1%, *n* = 2188). The utilization of the procedures also differed from ambulatory care settings. For example, gynecologic ultrasound was the most common procedure applied in medical centers, while pelvic examination was performed most frequently in physician clinics (Table 3). 

**Table 3:** Number of ambulatory visits to OB-GYNs (n = 35,697) by procedure from the 1/500 sampling datasets comprising 619,760 ambulatory visits in 2012, top 10.

NHI Code	Procedure	No. of visits	%
55021C	Pelvic examination	6,774	19.0%
33	Cervical pathology	4,536	12.7%
55011C	Vaginal irrigation	4,058	11.4%
31	Pap smear sampling/pelvic examination	3,653	10.2%
19003C	Gynecologic ultrasound	2,802	7.8%
06505C	Pregnancy test—enzyme immunoassays	2,188	6.1%
06012C	General urine examination	825	2.3%
19010C	Obstetric ultrasound	568	1.6%
41	First trimester prenatal visits	478	1.3%
64	Rubella IgG test	460	1.3%

Of the ambulatory visits to OB-GYNs, 55.4% (*n* = 19,760) were managed by prescribing medication. More than half the visits where medication was prescribed recorded prescriptions of two or fewer drugs. Approximately 87.7% of the visits where medication was prescribed recorded prescriptions of no more than two kinds of drugs. The most commonly prescribed medications were anti-infectives and antiseptics not in combination with corticosteroids (15.1%), anti-inflammatory and anti-rheumatic products, non-steroids (10.5%), and other analgesics and anti-pyretics (8.1%; Table 4).

**Table 4:** Number and percentage of ambulatory visits to OB-GYNs (n = 35,697), by prescribed drug therapeutic category, in Taiwan, from the 1/500 sampling datasets comprising 619,760 ambulatory visits in 2012, top 10.

ATC Code	Drug classification	No. of visits	%
G01A	Anti-infectives	5402	15.1%
M01A	Anti-inflammatory, non-steroids	3754	10.5%
N02B	Other analgesics and anti-pyretics	2884	8.1%
G03D	Progestogens	2871	8.0%
A02A	Antacids	2683	7.5%
D07C	Corticosteroids, combinations with antibiotics	2090	5.9%
R06A	Antihistamines for systemic use	1932	5.4%
A03A	Drugs for functional bowel disorders	1866	5.2%
J01D	Other beta-lactam anti-bacterials	1863	5.5%
G03C	Estrogens	1837	5.1%

## 4. Discussion

In this report, physician clinics remained the principal providers, receiving 61.6% (*n* = 21,974) of the ambulatory visits to OB-GYNs. On the other hand, free choice of physicians and facilities without referrals within Taiwan’s NHI might cause the OB-GYNs at hospital to take nearly two-fifths of the workload.

The shortage and aging of OB-GYNs are represented in Figure 2. Although the number of young female physicians has increased, the main workforce depends on male OB-GYNs aged 50–59 years. Because our analysis is based on actual claims from ambulatory visits, these doctors are really practicing physicians, not simply figureheads. Figure 2 indicates that older male physicians will soon retire. It is a top priority for healthcare authorities to find ways to close the gap. The reasons for shortage of new recruits include the decreasing birthrate, insufficient reimbursement and malpractice risk [4]. The government in Taiwan had already some childbirth encouragements such as childbirth subsidy, maternity pension and children education subsidy [11]. However, the decreasing birth rate had not been reversed [12]. Furthermore, measures to offer sufficient reimbursement and reduce malpractice risk for the OB-GYNs were still far from satisfactory.

In our study, the number of visits from patients aged over 60 years has noticeably dropped. Elderly patients no longer need to visit OB-GYNs for disorders of menstruation or pregnancy. However, osteoporosis and compression fractures are prevalent in this age group [13,14]. After menopause, women experience bone loss faster and encounter the morbidity earlier than men. According to a previous study in Taiwan [13], 50.3% of women aged 60–69 years, 63.7% of 70–79 years and 100% of 80–89 years had at least one BMD T-score ≤ −2.5. In another recent research in Taiwan, Hwang [14] also found many patients with osteoporotic fractures did not receive appropriate assessments or treatments. Although OB-GYNs can also manage osteoporosis, our results revealed the necessity to educate the public about early detections and interventions at OB-GYNs, especially for the peri- and postmenopausal women.

Incontinence is another disease worth mentioning. It is widely believed that stress or urgency incontinence is not uncommon in elderly women [15,16]. The prevalence was estimated to be up to 25% in women aged 50–65 in Taiwan, but only 27.1% of the patients sought medical help [15]. In our study, incontinence did not appear in the list of most frequent diagnoses in visits to OB-GYNs. It is unclear whether women underuse medical care for incontinence or visit other specialties instead [17].

In our study, anti-infective agents are the most frequently prescribed drugs. Probably most anti-infective agents are used to treat gynecological inflammatory diseases which rank third, ninth and tenth frequent diagnoses (Table 2). To decrease the overuse of antibiotics, the NHI in Taiwan has strictly regulated the prescription of antibiotics, especially for upper respiratory tract illness [18]. More investigations are required to evaluate the appropriateness of antibiotics prescribed by OB-GYNs. Besides, hormone therapy is not as popular in Taiwan as it is in the United States [2].

Our study with claims data from the National Health Insurance Administration has some limitations. First, the results do not include self-paid medicines or procedures, e.g., abortifacients or miscarriage surgery on demand. According to a survey by the Ministry of Health and Welfare in Taiwan, the incidence of artificial abortion among women of childbearing age is 1%–4.6% [19]. On the other hand, the more and more frequently practiced *in vitro* fertilization [20] is not yet reimbursed by Taiwan’s NHI. Second, the physicians made tentative diagnoses rather than final diagnoses for claims at ambulatory setting. The accuracy of the diagnoses can’t be guaranteed. Third, our analyses with visit-based sampling datasets can’t reveal the comorbidities and subsequent status of each patient.

## 5. Conclusions

In Taiwan, male OB-GYNs aged 50–59 years provided two-fifths of ambulatory care services. The aging of the country's health care manpower implies a shortage of new OB-GYNs entering the profession. In addition, the decreasing frequency of visits to OB-GYNs by elderly women and the high proportion of antibiotics prescribed during visits requires further study.

## References

[1] Ide H., Yasunaga H., Kodama T., Koike S., Taketani Y., Imamura T. (2009). The dynamics of obstetricians and gynecologists in Japan: A retrospective cohort model using the nationwide survey of physicians data. J. Obstet. Gynaecol. Res..

[2] Rayburn W.F., Strunk A.L., Petterson S.M. (2015). Considerations about retirement from clinical practice by obstetrician-gynecologists. Am. J. Obstet. Gynecol..

[3] American College of Obstetricians and Gynecologists. http://www.acog.org/Resources-And-Publications/Committee-Opinions/Committee-on-Health-Care-for-Underserved-Women/Health-Disparities-in-Rural-Women.

[4] Wang P.H., Sheu B.C., Yeh J.Y. (2009). The sunset industry: Obstetrics and gynecology concerns about the shortage of obstetricians and gynecologists. Am. J. Obstet. Gynecol..

[5] Ezzat T. (1978). Office visits to obstetrician-gynecologists, National Ambulatory Medical Care Survey, 1975. Adv. Data.

[6] Cypress B.K. (1984). Patterns of ambulatory care in obstetrics and gynecology: The national ambulatory medical care survey, United States, January 1980–December 1981. Vital Health Stat..

[7] Schappert S.M. (1993). Office visits to obstetricians and gynecologists, US 1989–90. Adv Data.

[8] Hill L.D., Erickson K., Holzman G.B., Power M.L., Schulkin J. (2001). Practice trends in outpatient obstetrics and gynecology findings of the Collaborative Ambulatory Research Network, 1995–2000. Obstet. Gynecol. Survey.

[9] Coleman V.H., Power M.L., Zinberg S., Schulkin J. (2004). Contemporary clinical issues in outpatient obstetrics and gynecology findings of the Collaborative Ambulatory Research Network 2001–2004 Part I. Obstet. Gynecol. Survey.

[10] Coleman V.H., Power M.L., Zinberg S., Schulkin J. (2004). Contemporary clinical issues in outpatient obstetrics and gynecology findings of the Collaborative Ambulatory Research Network 2001–2004 Part II. Obstet. Gynecol. Survey.

[11] The Guardian Taiwan Offers Baby Bonus to Fix Plummeting Birth Rate. http://www.theguardian.com/world/2012/jan/23/taiwan-low-birth-rate.

[12] Lee R., Mason A., members of the NTA Network (2014). Is low fertility really a problem? Population aging, dependency, and consumption. Science.

[13] Lin Y.C., Pan W.H. (2011). Bone mineral density in adults in Taiwan: Results of the Nutrition and Health Survey in Taiwan, 2005–2008 (NAHSIT 2005–2008). Asia Pac. J. Clin. Nutr..

[14] Hwang J.S., Chan D.C., Chen J.F., Cheng T.T., Wu C.H., Soong Y.K., Tsai K.S., Yang R.S. (2014). Clinical practice guidelines for the prevention and treatment of osteoporosis in Taiwan: Summary. J. Bone Miner Metab..

[15] Chen G.D., Lin T.L., Hu S.W., Chen Y.C., Lin L.Y. (2003). Prevalence and correlation of urinary incontinence and overactive bladder in Taiwanese women. Neurourol. Urodyn..

[16] Tseng L.H., Liang C.C., Lo H.P., Lo T.S., Lee S.J., Wang A.C. (2006). The prevalence of urinary incontinence and associated risk factors in Taiwanese women with lower urinary tract symptoms. Chang Gung Med. J..

[17] Chia P.C., Chou C.L., Chou Y.C., Shao C.C., Su H.I., Chen T.J., Chou L.F., Yu H.C. (2014). The ecology of gynecological care for women. Int. J. Environ. Res. Public Health.

[18] Huang N., Chou Y.J., Chang H.J., Ho M., Morlock L. (2005). Antibiotic prescribing by ambulatory care physicians for adults with nasopharyngitis, URIs, and acute bronchitis in Taiwan: A multi-level modeling approach. Fam. Pract..

[19] Department of Statistics, Ministry of Health and Welfare. http://www.mohw.gov.tw/cht/DOS/Statistic.aspx?f_list_no=312&fod_list_no=2218.

[20] Health Promotion Administration, Ministry of Health and Welfare. http://www.hpa.gov.tw/BHPNet/Web/Stat/StatisticsShow.aspx?No=200712250002.

